# Reforming China’s retail prescription medicine purchasing system: evidence from retail pharmacies in Wuhan

**DOI:** 10.1186/s12913-026-14233-7

**Published:** 2026-02-27

**Authors:** Zhihao Xu, Stephen Nicholas, Elizabeth Maitland, Jian Wang, Jie Li, Jialong Tan

**Affiliations:** 1 Dong Fureng Institute of Economic and Social Development, Wuhan University, Wuhan, Hubei Province China; 2 Health Services Research and Workforce Innovation Centre, Newcastle Business School University of Newcastle, Newcastle, NSW Australia; 3 Australian National Institute of Management and Commerce, Australian Technology Park, Sydney, NSW Australia; 4 School of Management, University of Liverpool, Liverpool, England, UK

**Keywords:** Insurance financing reform, Retail pharmacies, Medicines purchasing behavior, Interrupted time series analysis

## Abstract

**Background:**

In China, national health reforms are interpreted, piloted and implemented slightly differently by each local health authority. This is the first study of China’s 2023 pharmacy reform allowing 360 million urban employee basic medical insurance (UEBMI) members the right to purchase prescription medicine using their insurance at retail pharmacies. For Wuhan, we assess the impact of the 2023 medicine purchasing reform on medicine buying behavior; evaluate the benefits of the changes to UEBMI members; and provide new insights into China’s retail pharmacies’ business model.

**Methods:**

From March 2022 to December 2023, we gained unique access to daily UEBMI members’ medicine purchases, covering 34,956 claims at two representative Wuhan pharmacies. The impact of the new retail pharmacy purchasing policy on medicines purchasing behavior were quantitatively evaluated using descriptive statistics and interrupted time series analysis (ITSA).

**Results:**

No significant pre-reform trend in medicine purchases was observed, confirming a stable baseline prior to the 2023 reform. However, medicine purchases from retail pharmacies showed a significant upward trend. There was no evidence of medicine over-use or fraud. The benefit to members was measured by the absence of any significant upward per capita total expenditure trend and a significant decrease in the proportion of medicine expenditures from members personal medical savings accounts. Retired UEBMI members benefited more than currently employed UEBMI members from allowing UEBMI funds for prescription purchases from retail pharmacies.

**Conclusions:**

The 2023 reforms allowed UEBMI members to purchase medicine from retail pharmacies, providing medicine accessibility and affordability for members, especially retired members; did not trigger an increase in members’ medicine consumption; attenuated the over-use of hospital pharmacies. Future study should empirically test whether pharmacies’ business model transformed away from a singular focus on price competition towards a combination of price competition and health services.

## Background

In February 2023, China reformed its prescription medicine purchasing system. Retail pharmacy reluctance to share private purchasing and pricing data explains the absence of studies on the 2023 pharmacy reform in China. Pre-reform, purchasing medicines in retail pharmacies and hospital pharmacies was different. Regulated by the pharmacy administration committee, most medicines in hospital pharmacies targeted serious diseases, while most medicines in retail pharmacies were regulated by private owners for common diseases. For a range of diseases, retail and hospital pharmacies dispensed the same medicines. Hospital prescription medicines were covered by the the social pooling account (SPA) of China’s urban employee basic medical insurance (UEBMI), while the expenditures of medicines in retail pharmacies were paid by out-of-pocket (OOP) cash or UEBMI member’s private medical savings accounts (MSAs). China’s 2023 retail pharmacy reform allowed 360 million UEBMI members to use their UEBMI insurance to make prescription medicine purchases at retail pharmacies. As a template for the reform of prescription medicine purchases in China, we assess the impact of Wuhan’s 2023 medicine purchase reform policy on the medicine purchasing behavior of UEBMI members, evaluate the benefits of the changes to members, and provide recommendations for China’s retail pharmacies business model.

Since the establishment of China’s UEBMI in 1998, social medical health insurance has been a fundamental element in China’s healthcare security system [1, 2]. Funded by employed members and their employers, UEBMI is the largest social medical insurance plan in China covering over 360 million urban employed and retired workers, or over 25% of the population. Employed members become lifetime members after paying the annual premium for 25 years for men and 20 years for women. The premium paid by working members is 2% of the member’s salary, deposited into the individual member’s MSAs, while the premium paid by employers, which is 6% of the member’s salary, was partly deposited into MSAs (30%) and partly into SPA (70%). The SPA is administered by the local healthcare security administration for inpatient services and outpatient services for illnesses. Pre-2023 reform, MSAs were mainly used for outpatient services, but post-2023 reform, both MSAs and SPA were used for outpatient services and medicine purchasing in retail pharmacies. UEBMI’s reimbursable coverage of medicines, medical consumables and facility use is regulated by the National Reimbursement Drug List (NRDL), National Reimbursement Medical Consumables List (NRMCL) and National Reimbursement Medical Service Facility List (NRMSFL). Like other types of health insurance, UEBMI patients must pay a deductible (approximately 10% of average annual wages of a local urban worker) for health services and medicine, with the remainder is shared between SPA (usually 70–80%) and MSA (usually 20–30%). When the maximum of SPA (fourfold local average annual wages) has been paid or an individual’s MSA has been exhausted, the patient must pay their expenses as OOP cash payments. Unspent funds in MSAs can be carried forward to the next year, and any remaining balance of a member’s MSA at death can become part of their estate [3]. In 2022, RMB2079.33 billion (US$287.36 billion) was deposited into SPA and RMB763.31 billion (US$105.49 billion) into MSAs.

In hospital pharmacies, UEBMI’s SPA covered medicine expenditures as part of inpatient or outpatient services. Expenditures of medicines in retail pharmacies were not covered by UEBMI SPA, but paid by OOP cash or MSAs. To avoid medicine price markups by hospitals, the prices of medicines in hospital pharmacies were required to be equivalent to their purchase cost [4]. Prices of medicines in retail pharmacies were decided by market competition between privately owned pharmacies. Similar to Singapore’s mediSave account system [5], UEBMI’s SPA arrangements restricted the expansion of outpatient healthcare coverage as healthcare demands have increased [6]. UEBMI members, especially retired members with serious or multiple chronic diseases, faced marked difficulties in affording medicines and outpatient healthcare services not covered by SPA, but dispensed by retail pharmacies [7]. Second, the lack of UEBMI’s SPA for retail pharmacy outpatient services led to excessive hospitalization and wasteful over-use of hospital resources, including medicines. The 2019 hospitalization rate of the UEBMI members was 18.7% [8], much higher than countries and regions with a similar GDP level to China and some OECD countries [9]. Finally, China’s unequal development meant that MSA balances for outpatient and medicine purchases impacted UEBMI members differently depending on members’ provinces and regions, with deficits in less developed and excessive balances in developed areas [10].

**Fig. 1:**
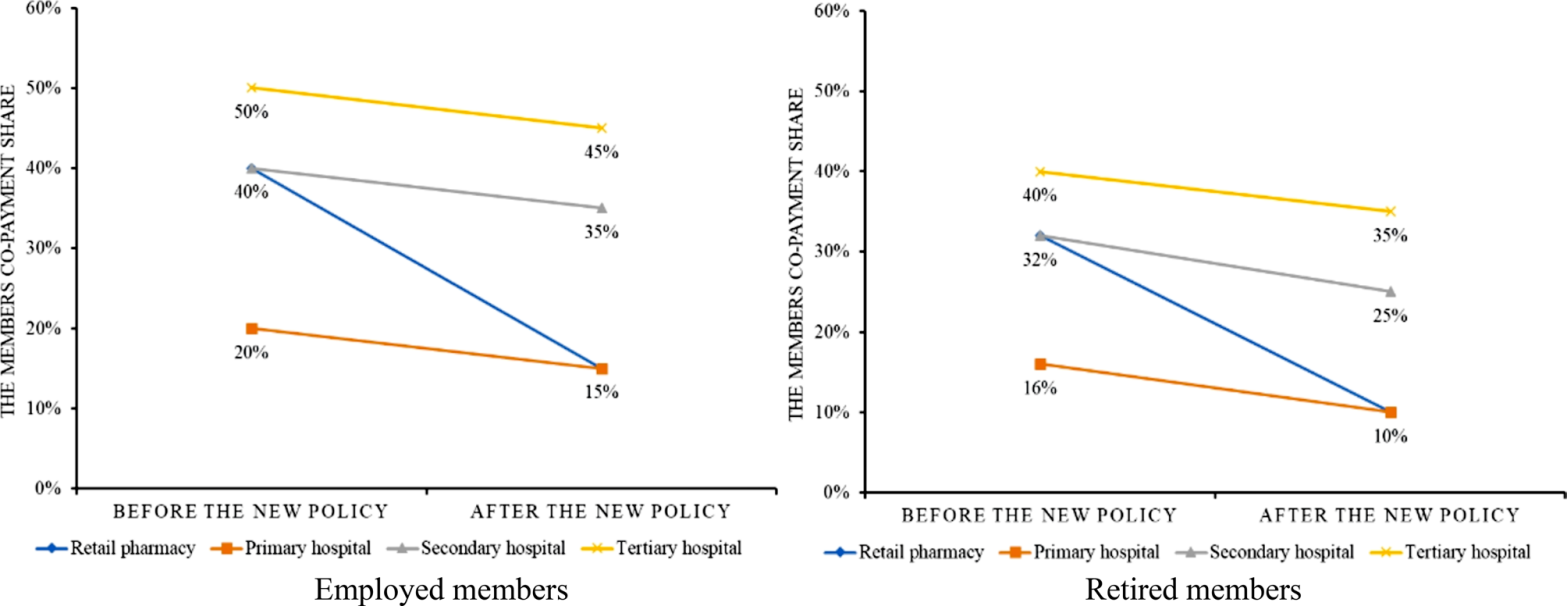
Changes in the members co-payment share after the 2023 reform policy

To address issues specific to retail pharmacy medicine purchases, the government issued a new policy in 2023 to include eligible retail pharmacies in the UEBMI’s SPA outpatient scheme [11]. Each local and regional health authority have interpreted, piloted and implemented the new policy slightly differently. On February 13, 2023, Wuhan issued a new local UEBMI policy stating that both SPA and MSA were available for medicines prescribed by outpatient physicians in hospitals and purchased in retail pharmacies. After the deductible, Fig. 1 shows the changes to the co-payment share by employed and retired members for medicines at Wuhan’s retail pharmacies, primary hospitals, secondary hospitals and tertiary hospitals. In all cases, the co-payments fell, benefiting members, with the co-payment for retail pharmacies pegged at the same level as primary hospitals. As part of the reforms, the deductible, previously RMB700 (US$96.74) for employed members and RMB500 (US$69.10) for retired members, was eliminated; the annual reimbursement maximum for retired members was increased from RMB4000 (US$552.80) to RMB4500 (US$621.90). Finally, all the insurance scheme coverage expansion mentioned above came from the increase of SPA, driven by the 30% reallocation of employer premiums from MSAs to SPA.

Similar to other reforms [12–14], the 2023 changes to UEBMI health insurance coverage aimed to increase the utilization of retail pharmacy healthcare services, promote better member health outcomes and control the over-use of hospital health resources. But research on the impact of the 2023 UEBMI changes to medicine purchasing in retail pharmacies on members and retail pharmacies is scarce, mainly due to limited access to data on UEBMI members’ medicine purchases. How did Wuhan’s 2023 medicine purchase reforms change UEBMI members medicine purchasing behavior?; Who benefited?; and What were the challenges to China’s retail pharmacies’ business model?

**Fig. 2:**
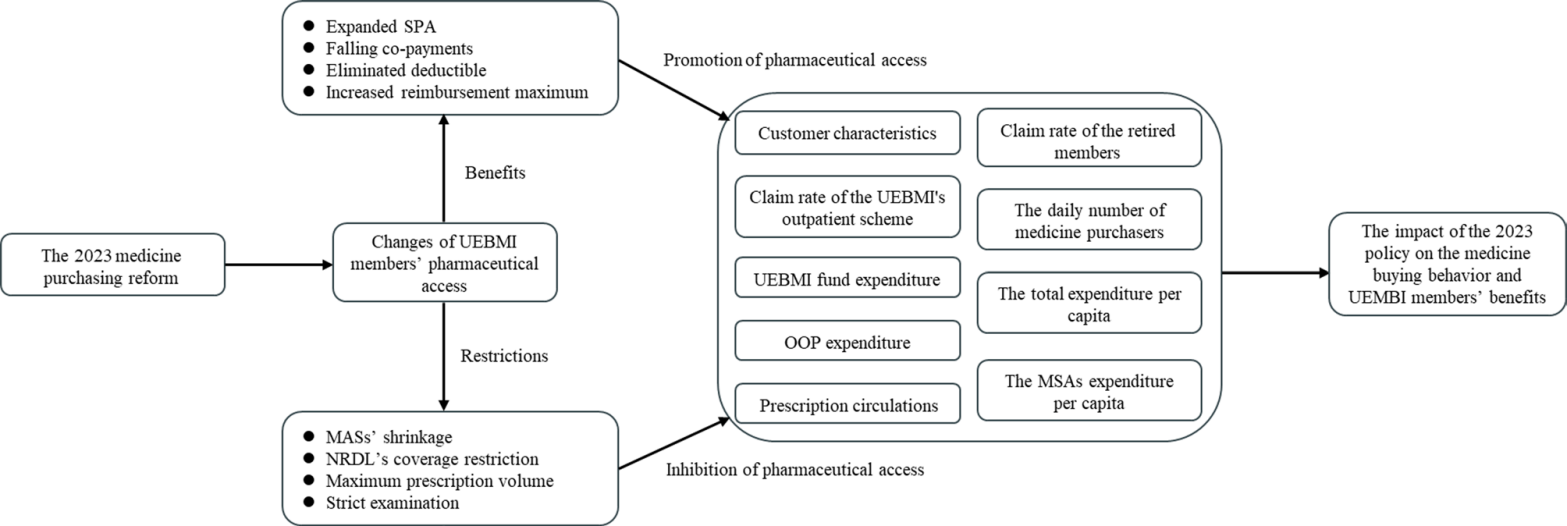
Conceptual model

Figure 2 sets out a framework for investigating the impact of the 2023 policy on the medicine buying behavior and UEMBI members’ benefits. The 2023 reforms changed UEBMI members’ pharmaceutical benefits and restrictions, and members’ medicine buying behavior. Among the benefits, the SPA pooling fund was expanded, with 30% of the premium paid by employers deposited to SPA instead of members’ individual MSA. Falling co-payments, eliminating the deductible and increasing the reimbursement maximum were the other core reform elements. The 2023 reforms also included SPA management and claim risk restrictions. First, the expanded SPA meant the MSA shrinkage, with less balances to pay OOP retail pharmacy medicine expenditures. Second, medicines in the NRDL’s coverage were now available for SPA reimbursement, while previously they were paid as OOP expenses or MSA. Third, the maximum prescription volume was not allowed to exceed three days’ usage, which prevent SPA from paying for medicines to be hoarded or resold. Finally, when purchasing medicine recorded in NRDL, whether prescription or over-the-counter (OTC) drugs, the members had to provide both individual and prescriptions information, which formerly applied only for prescription drugs.

Figure 2 also shows the reform’s promotion and inhibitions to pharmaceutical access through benefits and restrictions. The 2023 reform benefits might mean more UEBMI members, especially retired ones, utilizing the UEBMI’s outpatient scheme in retail pharmacies, consuming more, or more expensive, medicines, leading to higher individual and SPA expenditures. On the other hand, the MSA shrinkage and maximum prescription volume potentially inhibited UEBMI medicine consumption. To make the reform’s direction of influence clear, our study measured and examined the changes in a series of outcome indicators, shown on the right-hand side of Fig. 2, which include variables such as the number of insurance claims, expenditures, and reimbursements. These indicators serve as dependent variables reflecting the reform’s impact on medicine-buying behavior and UEBMI members’ benefits.

## Methods

### Data and measures

Given competition between China’s retail pharmacies and their private ownership, pharmacies were reluctant to allow access to private purchasing and pricing data, which explains the absence of studies on the 2023 pharmacy reform in China. We have developed a unique dataset of UEBMI retail pharmacy prescription medicine purchases. For academic study and under strict confidentiality rules, one of Wuhan’s leading pharmacy chains granted access to individual medicine claim data at Pharmacy A, located in a residential community near several hospitals, and Pharmacy B, located in a commercial pedestrian street in a business district. The two retail pharmacies represent the main geographical distribution of retail pharmacies either near hospitals, business districts or residential communities. This chain operates more than twenty retail pharmacies in Wuhan, and the annual revenue, number of employees, space and number of medicines on sale in the two pharmacies were close to the average level of all pharmacies in Wuhan.

Following the implementation of Wuhan’s local UEBMI policy on February 13, 2023, Pharmacies A and B were incorporated into the new UEBMI outpatient reimbursement scheme. No other major policy, regulatory, or market events were identified during the study period that could plausibly affect our sample pharmacies around the intervention time, suggesting that the observed changes are unlikely to be confounded by concurrent external factors. We collected daily UEBMI claim data from March 1, 2022, to December 15, 2023. The data involved customer information, medicines purchased and claim information by UEBMI members who purchased medicines and received reimbursed through UEBMI’s outpatient scheme. Based on previous studies on health service utilization [15, 16], we evaluated policy effects from two facets: customer characteristics and medicine purchasing behavior. Customer characteristics included age, sex, employment status, claim type and the source of prescriptions. Employment status was currently employed UEBMI members or retired members, with the UEBMI’s outpatient scheme for retired members more generous than for the currently employed [17]. The claim type was whether the expenditure was reimbursed by the UEBMI’s outpatient scheme at the same rate as hospital pharmacies. The source of prescriptions was classified as whether the medicine purchaser’s UEBMI was enrolled in Wuhan, and whether the medicines were prescribed by the physician at a hospital as part of the prescription circulation scheme. Prescription circulation connected prescription data between hospitals and retail pharmacies, where physicians in hospitals upload their electronic prescriptions to the platform accessed by retail pharmacies allowing patients to purchase medicines from any eligible retail pharmacy. Prescription circulation aimed to address the over-use of hospital pharmacies, increase hospital—retail pharmacy competition, benefit UEBMI members by easing access to medicine through retail pharmacies, and strengthen the supply capacity of retail pharmacies [18].

The number of medicine purchasers (*NMP*), the total expenditure per capita ($TE_{pc}$), and the MSAs expenditure per capita ($\:{ME}_{pc}$) were used to measure the medicine purchasing behavior and their changes before and after the implementation of the 2023 policy. $\:NMP$ was the total number of medicine purchases measured in days, and $\:{TE}_{pc}$ and $\:{ME}_{pc}$ were the total and average expenditure measured in days, respectively. Retail pharmacy hours were similar to hospital pharmacy hours.

### Empirical model

In common with previous research [19, 20], descriptive statistics, comprising mean, standard deviation and t-test, were employed to analyze the customer characteristics. Chi-square tests were employed for disordered classified data, and the difference was statistically significant at *P* < 0.05. Interrupted time series analysis (ITSA) [21] was used to assess the longitudinal effects of including retail pharmacies in UEBMI’s outpatient medicine purchasing scheme. We used the Durbin-Watson (DW) statistic to detect the residual autocorrelation in the time series data. We fitted regression models for the two periods before and after the implementation of the reform:

$$\:{Y}_{t}=\alpha\:+{\beta\:}_{1}{X}_{1}+{\beta\:}_{2}{X}_{2}+{\beta\:}_{3}{X}_{3}+{\varepsilon\:}_{t}$$

where $\:{Y}_{t}$ is the outcome variables $\:NMP$, $\:{TE}_{pc}$ or $\:{ME}_{pc}$ of day $\:t$; $\:{X}_{1}$ is a continuous time variable, representing the time since the beginning of the study. Given the initial publicity after the reform was implemented, our study assumed that the intervention time of the reform was March 1, 2023 which is represented by $\:{X}_{1}$=1, and the ending time of December 15, 2023 is represented by $\:{X}_{1}$=652; $\:{X}_{2}$ is the intervention variable which is 0 before and 1 after the reform was implemented; $\:{X}_{3}$ is a continuous variable, representing the number of days since the reform was implemented. December 15, 2023 was represented by $\:{X}_{3}$=319, and $\:{X}_{3}$=0 or =$\:{X}_{1}$-319, to represent the day before and after the reform was implemented. In our model, α is the estimated baseline level of $\:NMP$, $\:{TE}_{pc}$ or $\:{ME}_{pc}$; $\:{\beta\:}_{1}$ is the estimated slope of the outcome variables before the reform was implemented; $\:{\beta\:}_{2}$ is the estimated level change of the outcome variables after the intervention; $\:{\beta\:}_{3}$ is the estimated slope change of the outcome variables after the intervention; $\:{\epsilon\:}_{t}$ is the error term, indicating random effects that the model cannot explain.

## Results

### Customer characteristics

Table 1 summarizes the 12,772 claims extracted from Pharmacy A and Table 2 22,184 claims from Pharmacy B. In terms of the age, sex and employment status, the reform did not lead to any statistically significant changes in UEBMI members’ characteristics. The customers remained mainly middle-aged and older members, with the majority current employees, indicating that the demand for medicines did not change significantly with the 2023 reform. However, the number of prescription circulations increased on average by 30% after the implementation of the 2023 reform, which means that patients increasingly chose to purchase medicines at retail pharmacies after their medicines were prescribed by hospital doctors.

After the implementation of the 2023 reform, the total expenditure per capita for members in the two pharmacies fell by RMB33.02 (US$4.56) in Pharmacy A and RMB21.41 (US$2.96) in Pharmacy B. From 100%, the average out-of-pocket proportion was reduced to 78.99% in Pharmacy A and 80.10% in Pharmacy B, which shows that the reform helped relieve the out-of-pocket economic burden on members. These price changes were driven by the average UEBMI SPA expenditure, which changed from no contribution to RMB18.66 (US$2.58) per capita in Pharmacy A and RMB14.21 (US$1.96) per capita in Pharmacy B insurance contribution. While the 2023 reform cancelled the transfer of 30% employer premiums to MSA, the MSAs expenditure per capita was reduced by RMB50.35 yuan (US$6.96) in Pharmacy A and RMB35.87 yuan (US$4.96) in Pharmacy B.

**Fig. 3:**
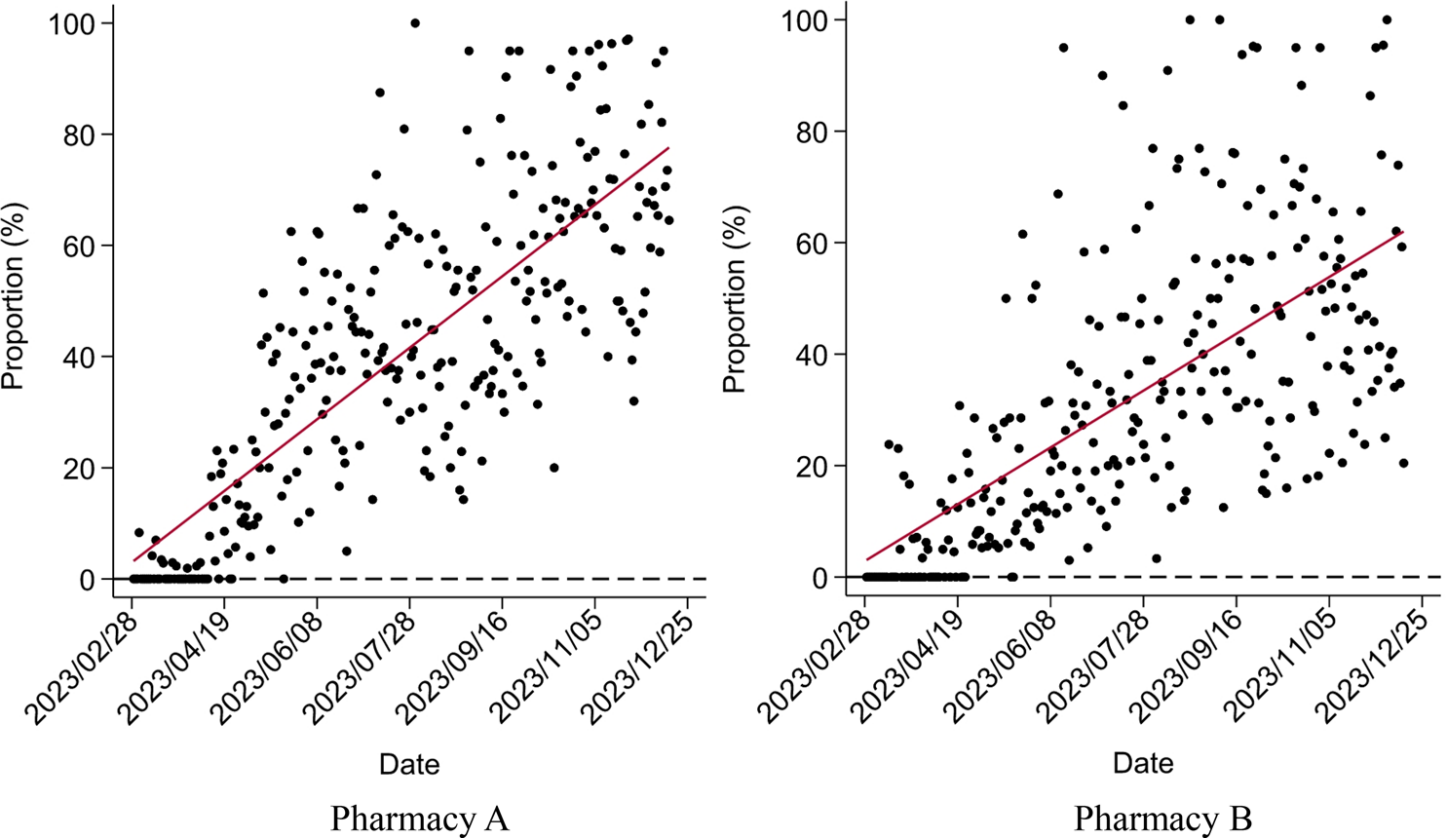
Changes in the proportion of members claimed by the UEBMI’s outpatient scheme per day after the new policy

**Fig. 4:**
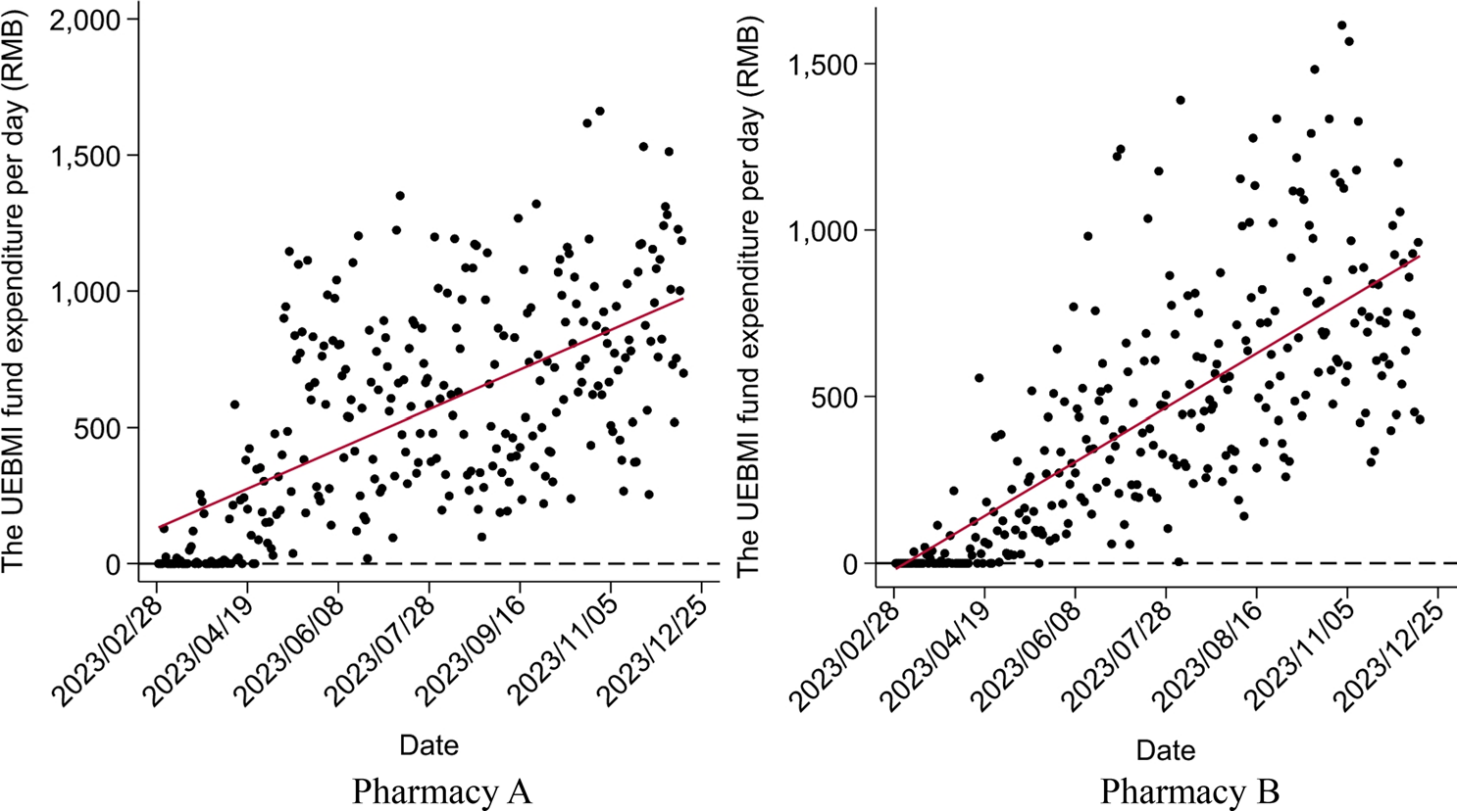
Changes in the UEBMI fund expenditure per day after the new policy

**Fig. 5:**
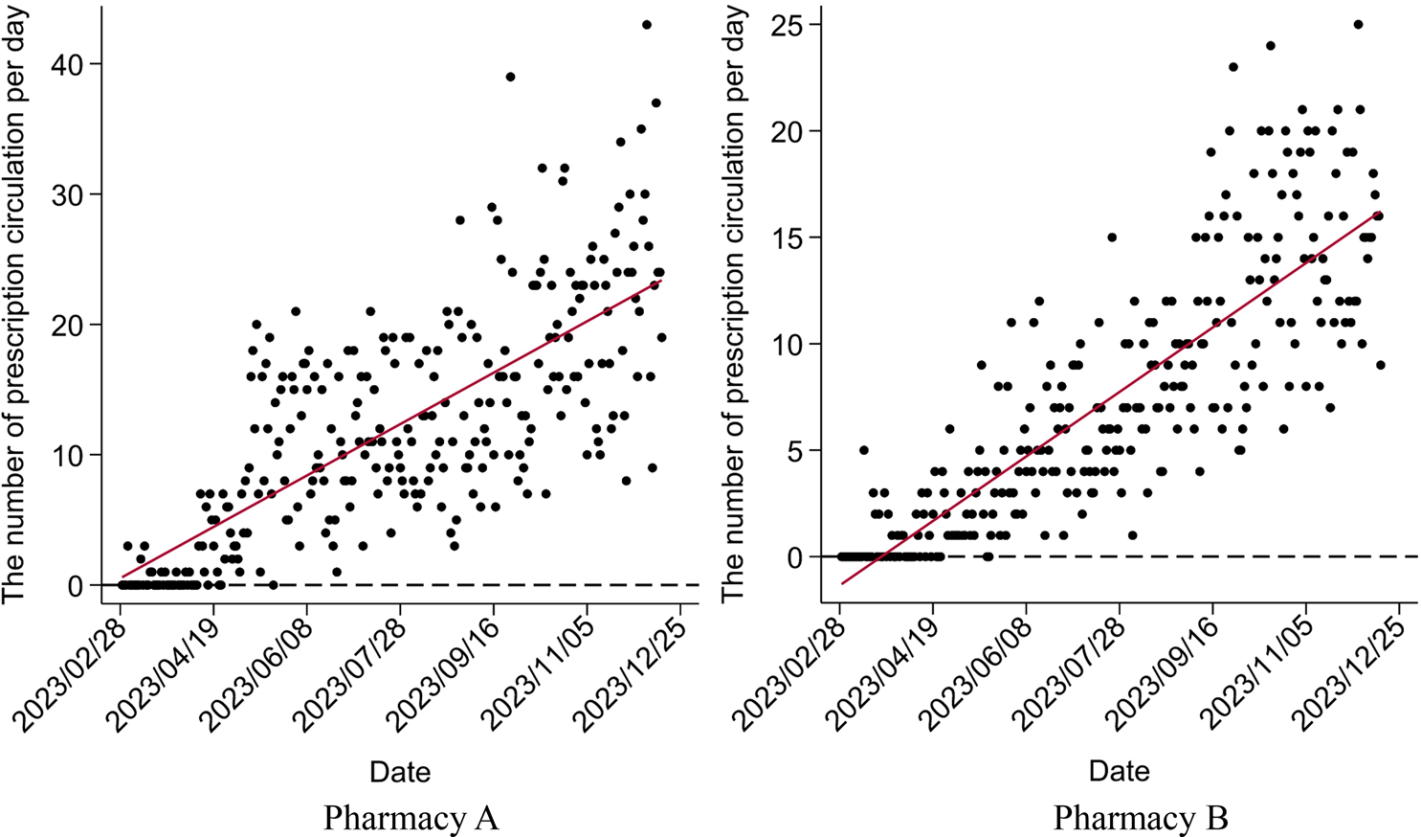
Changes in the number of prescription circulations per day after the new policy

Although both pharmacies were included in UEBMI’s outpatient scheme in early February 2023, customers did not claim through this channel until late February. Figure 3 shows that as an increasing number of patients learned about the reform, the daily proportion of customers claiming through UEBMI’s outpatient scheme in both pharmacies continually increased, which meant a significant increase in UEBMI SPA expenditures in both pharmacies in Fig. 4. Figure 5 shows the implementation of the 2023 reforms encourage patients to switch their medicine purchasing from hospitals to retail pharmacies, promoting the use of the prescription circulation system.

The Chi-square test in Table 3 revealed a significant difference in the UEBMI’s outpatient scheme utilization rate between employed and retired members (χ²=124.759, *P* < 0.001 for Pharmacy A; χ²=118.245, *P* < 0.001 for Pharmacy B). The more generous coverage for retired members worked as an effective economic incentive, leading to higher retail pharmacy utilization rate by retired members. Given that most retired members suffer from more chronic diseases, the reform played a significant role in reducing the long-term financial burden on retired members.

**Table 1:** Descriptive statistics of customer characteristics for Pharmacy A

Variables	Before (*n* = 5342) Mean ± SD or *n* (%)	After (*n* = 7430) Mean ± SD or *n* (%)	T or χ² value	*P*-value
**Sex**				
Male	2592(48.52%)	3592(48.34%)	0.039	0.844
Female	2750(51.48%)	3838(51.66%)		
**Employment status**				
Employed	4476(83.79%)	6306(84.87%)	2.773	0.096
Retired	866(16.21%)	1124(15.13%)		
**Claim type**				
UEBMI’s outpatient scheme	0(0%)	2180(29.34%)	1889.960	< 0.001
Others*	5342(100%)	5250(70.66%)		
**Source of prescriptions**				
Not registered in Wuhan	51(0.95%)	590(7.94%)	3225.916	< 0.001
Registered in Wuhan	5291(99.05%)	4671(62.87%)		
Prescription circulation	0(0%)	2169(29.19%)		
**Age**	42.23 ± 14.52	42.69 ± 13.45	1.358	0.463
$\:{\boldsymbol{T}\boldsymbol{E}}_{\boldsymbol{p}\boldsymbol{c}}$	121.85 ± 237.03	88.83 ± 157.52	128.95	< 0.001
**OOP expenditure**	121.81 ± 237.04	70.17 ± 155.23	202.14	< 0.001
**UEBMI fund expenditure**	0.04 ± 1.4	18.66 ± 43.14	-239.03	< 0.001
$\:{\boldsymbol{M}\boldsymbol{E}}_{\boldsymbol{p}\boldsymbol{c}}$	119.55 ± 216.36	69.2 ± 154.83	203.30	< 0.001
**Cash expenditure**	2.25 ± 89.64	0.97 ± 10.63	9.49	< 0.001

Note: UEBMI, Urban Employee Basic Medical Insurance,$\:{\mathrm{T}\mathrm{E}}_{\mathrm{p}\mathrm{c}}$, the total expenditure per capita, OOP, out-of-packet, ME_pc_, the MSAs expenditure per capita; *Customers are not reimbursed for medicines that are not on the National Reimbursement Drug List (NRDL)

**Table 2:** Descriptive statistics of customer characteristics for Pharmacy B

Variables	Before (*n* = 11083) Mean ± SD or *n* (%)	After (*n* = 11101) Mean ± SD or *n* (%)	T or χ² value	*P*-value
**Sex**				
Male	4984(44.97%)	4876(43.92%)	2.457	0.117
Female	6099(55.03%)	6225(56.08%)		
**Employment status**				
Employed	10,507(94.80%)	10,549(95.03%)	0.580	0.446
Retired	576(5.20%)	552(4.97%)		
**Claim type**				
UEBMI’s outpatient scheme	0(0%)	3537(31.86%)	4201.082	< 0.001
Others*	11,083(100%)	7564(68.14%)		
**Source of prescriptions**				
Not registered in Wuhan	270(2.44%)	859(7.74%)	4896.234	< 0.001
Registered in Wuhan	10,803(97.66%)	6756(60.86%)		
Prescription circulation	0(0%)	3486(31.40%)		
**Age**	35.69 ± 10.43	35.75 ± 10.26	0.254	0.846
$\:{\boldsymbol{T}\boldsymbol{E}}_{\boldsymbol{p}\boldsymbol{c}}$	94.91 ± 148.71	73.5 ± 130.45	134.95	< 0.001
**OOP expenditure**	94.75 ± 148.59	58.87 ± 130.05	226.37	< 0.001
**UEBMI fund expenditure**	0.04 ± 2.59	14.21 ± 30.23	-260.59	< 0.001
$\:{\boldsymbol{M}\boldsymbol{E}}_{\boldsymbol{p}\boldsymbol{c}}$	94.38 ± 147.77	58.51 ± 129.78	226.75	< 0.001
**Cash expenditure**	0.37 ± 15.09	0.36 ± 6.19	0.23	0.268

Note: UEBMI, Urban Employee Basic Medical Insurance,$\:{\mathrm{T}\mathrm{E}}_{\mathrm{p}\mathrm{c}}$, the total expenditure per capita, OOP, out-of-packet, ME_pc_, the MSAs expenditure per capita; *Customers are not reimbursed for medicines that are not on the National Reimbursement Drug List (NRDL)

**Table 3:** Chi-square test of the claim rate of employed and retired members

Pharmacy	Employment state	UEBMI’s outpatient scheme *n* (%)	Others* n (%)	χ² value	*P*-value
Pharmacy A	Employed	1668(15.47%)	9111(84.53%)	124.759	< 0.001
	Retired	512(25.73%)	1478(74.27%)		
Pharmacy B	Employed	3302(15.68%)	17,754(84.53%)	118.245	< 0.001
	Retired	254(25.73%)	874(74.27%)		

*Customers are not reimbursed for medicines that are not on the National Reimbursement Drug List (NRDL)

### Medicine purchasing behavior

**Fig. 6:**
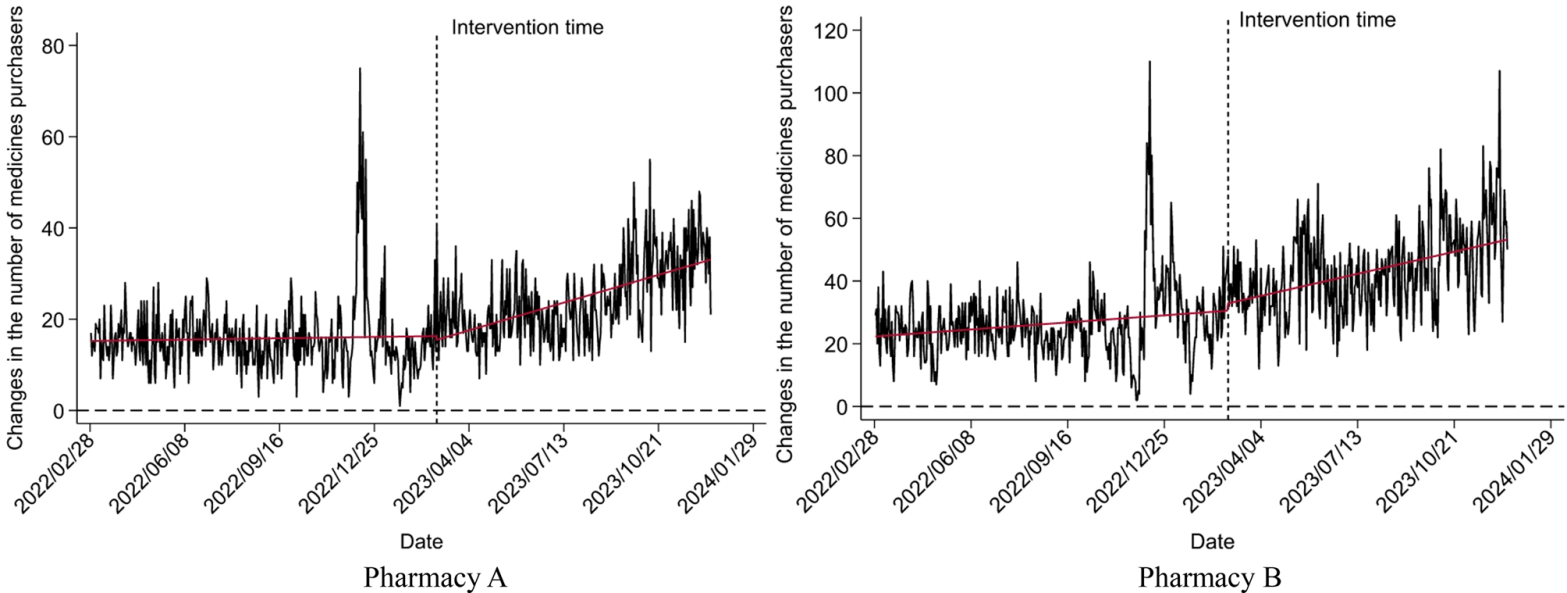
Changes in $\:NMP$ after the new policy using ITSA

**Fig. 7:**
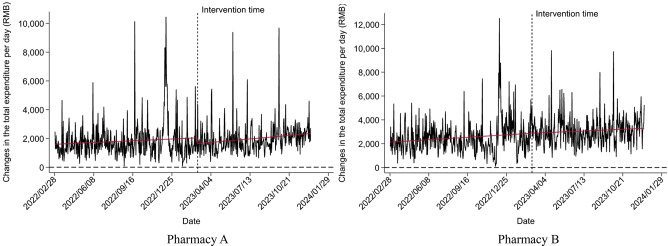
Changes in $\:{TE}_{pc}$ after the new policy using ITSA

**Fig. 8:**
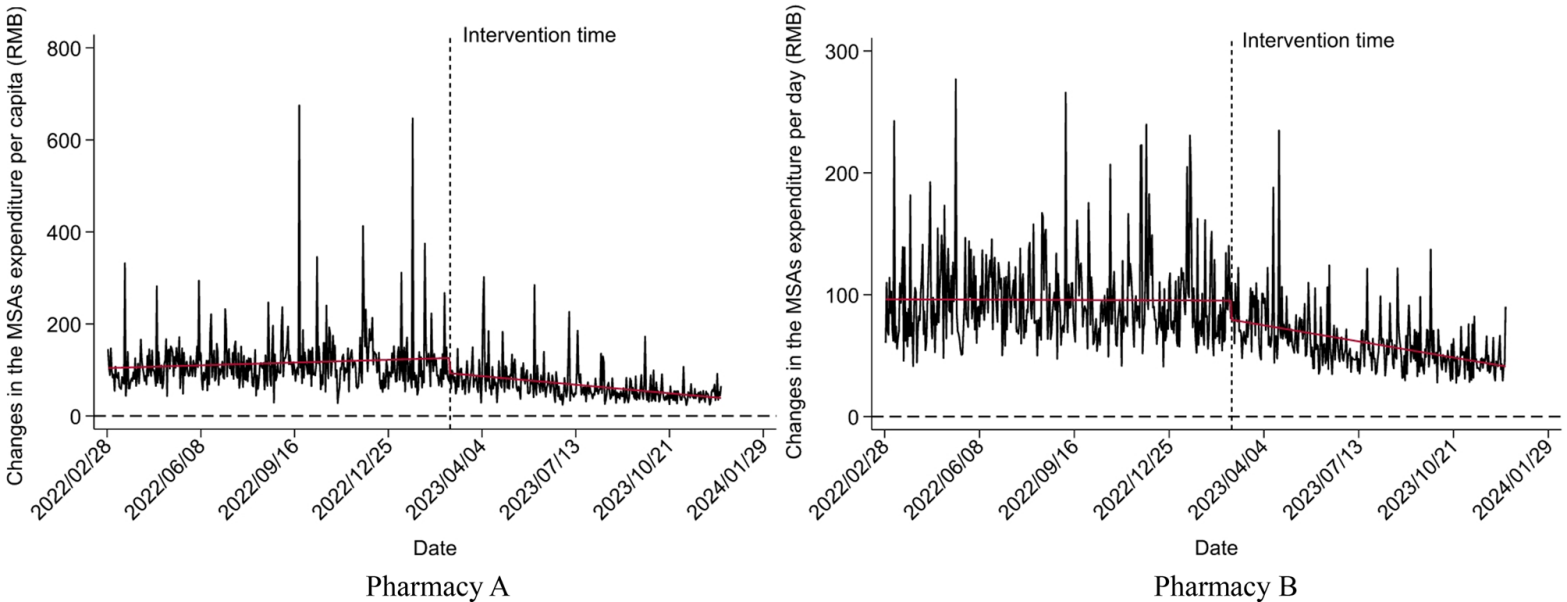
Changes in $\:{ME}_{pc}$ after the new policy using ITSA

The results of medicine purchasing behavior using ITSA are presented in Tables 4, 5 and 6; Figs. 6, 7 and 8. The Durbin-Watson (DW) statistics of the three outcome variables, $\:NMP\:2.186$, $\:{TE}_{pc}$ 2.085, and $\:{ME}_{pc}$ 1.999, indicate that there was no autocorrelation in the model residuals. Before the implementation of the reform, the $\:NMP$ of Pharmacy A (*P* = 0.756) and $\:{TE}_{pc}$ (*P* = 0.140) showed a non-significant increasing trend. In the month of intervention, changes in both $\:NMP$ (*P* = 0.943), and $\:{TE}_{pc}$ (*P* = 0.151) were also not statistically significant. After the reform, $\:NMP$ increased significantly, with a slope of 0.058 ($\:{\beta\:}_{1}$+$\:{\beta\:}_{3}$=0.002+0.056, *P* < 0.001), but the increase of $\:{TE}_{pc}$ was not statistically significant, with a slope of 2.242 ($\:{\beta\:}_{1}$+$\:{\beta\:}_{3}$=1.169+1.073, *P* = 0.431). In addition, $\:{ME}_{pc}$ showed an upward trend before the reform (*P* = 0.014), but significantly declined after, with a slope of -0.186 ($\:{\beta\:}_{1}$+$\:{\beta\:}_{3}$=0.065–0.251, *P* < 0.001).

The situation at Pharmacy B was slightly different. Before the implementation of the reform, $\:NMP$ showed an increasing trend (*P* = 0.049) which increased after the reform, with a significant slope of 0.067 ($\:{\beta\:}_{1}$+$\:{\beta\:}_{3}$=0.020+0.047, *P* = 0.007). Although $\:{TE}_{pc}$ increased in the month of intervention (*P* = 0.559), the increase was not significant after the reform with a slope of 1.004 ($\:{\beta\:}_{1}$+$\:{\beta\:}_{3}$=1.757 − 0.7533, *P* = 0.609). Different from Pharmacy A, $\:{ME}_{pc}$ of Pharmacy B showed a downward non-significant trend before the reform (*P* = 0.757), but a significant downward trend after the reform with a slope of -0.139 ($\:{\beta\:}_{1}$+$\:{\beta\:}_{3}$=-0.005-0.134, *P* < 0.001). This suggests that in the early stage of the reform, the members did not know or understand the new purchasing policy, and it took time for them to adapt to the reform. However, after the policy adaptation period, the reform achieved positive effects, attracting more members to retail pharmacies, but without any significant increase in the total expenditure per capita.

**Table 4:** The impact of the new policy on $\:NMP$ based on ITSA

Pharmacies	Coefficients	Values	Std.	T value	*P*-value
Pharmacy A	Constant	15.415	1.220	12.635	< 0.001
	Baseline slope $\:{\beta\:}_{1}$	0.002	0.006	0.311	0.756
	Step slope$\:\:{\beta\:}_{2}$	-0.129	1.808	-0.071	0.943
	Slope change$\:\:{\beta\:}_{3}$	0.056	0.010	5.634	< 0.001
Pharmacy B	Constant	22.628	2.132	10.615	< 0.001
	Baseline slope $\:{\beta\:}_{1}$	0.020	0.010	1.971	0.049
	Step slope$\:\:{\beta\:}_{2}$	3.352	3.133	1.070	0.285
	Slope change$\:\:{\beta\:}_{3}$	0.047	0.017	2.726	0.007

**Table 5:** The impact of the new policy on $\:{TE}_{pc}$ based on ITSA

Pharmacies	Coefficients	Values	Std.	T value	*P*-value
Pharmacy A	Constant	1608.262	165.859	9.697	< 0.001
	Baseline slope $\:{\beta\:}_{1}$	1.169	0.791	1.478	0.140
	Step slope$\:\:{\beta\:}_{2}$	-354.986	246.973	-1.437	0.151
	Slope change$\:\:{\beta\:}_{3}$	1.073	1.360	0.789	0.431
Pharmacy B	Constant	2162.662	181.560	11.912	< 0.001
	Baseline slope $\:{\beta\:}_{1}$	1.757	0.866	2.028	0.043
	Step slope$\:\:{\beta\:}_{2}$	157.860	269.783	0.585	0.559
	Slope change$\:\:{\beta\:}_{3}$	-0.753	1.474	-0.511	0.609

**Table 6:** The impact of the new policy on $\:{ME}_{pc}$ based on ITSA

Pharmacies	Coefficients	Values	Standard error	T value	*P*-value
Pharmacy A	Constant	103.857	5.518	18.823	< 0.001
	Baseline slope $\:{\beta\:}_{1}$	0.065	0.026	2.456	0.014
	Step slope$\:\:{\beta\:}_{2}$	-34.571	8.253	-4.189	< 0.001
	Slope change$\:\:{\beta\:}_{3}$	-0.251	0.045	-5.543	< 0.001
Pharmacy B	Constant	96.495	3.574	26.999	< 0.001
	Baseline slope $\:{\beta\:}_{1}$	-0.005	0.017	-0.309	0.757
	Step slope$\:\:{\beta\:}_{2}$	-13.453	5.326	-2.526	0.012
	Slope change$\:\:{\beta\:}_{3}$	-0.134	0.029	-4.609	< 0.001

## Discussion

This is the first empirical study of China’s 2023 retail pharmacy medicine purchasing reform on UEBMI members’ medicine buying behavior. Our results show that the 2023 reform had a positive impact on medicine accessibility and affordability, with the electronic circulation prescription system promoting medicine purchasing in the retail pharmacies by UEBMI members. Direct retail pharmacy medicine purchasing greatly reduced the registration and waiting time in hospitals, when members did not need any diagnosis and examination in hospital outpatient departments. Retail pharmacy purchases attenuated the over-utilization of hospital resources and increased medicine purchasing convenience of members. The 2023 reforms supported members’ retail pharmacy medicine purchases not only by eliminating the deductible portion, especially for retired members, but also by providing members with a generous reimbursement level from SPA as well as MSA.

The reform increased the medicine purchasing satisfaction of members and their loyalty to the retail pharmacy’s brand. Since attending hospital pharmacies imposed time and economic costs on UEBMI members, the new policy especially benefited members suffering from chronic diseases, common diseases and frequently-occurring diseases by eliminating the need for frequent visits to hospital pharmacies. Another benefit for members was the availability of preliminary diagnosis services and recommendations for medicines by health consultants at retail pharmacies. These retail pharmacies services also increased patient trust in the retail pharmacies close to members’ homes, encouraging return visits. A further benefit for patients was to attenuate the vicious competition between different retail pharmacies in terms of price by gradually shifting pharmacies away from price competition towards competition in professional health services to attract more UEBMI members. This change significantly transformed retail pharmacies’ business models, focusing retail pharmacy strategy on improving professional health services, such as health consultation and chronic disease management, and meeting customers’ future health demands [22].

The ITSA results showed no increase in the consumption of medicines after the 2023 reform, which contrasts with the results of studies on different types of insurance changes in China [23–25]. There are several individual-level reasons for the absence of increases in medicine consumption after the 2023 reform. Only part of the retail pharmacy medicine purchases were covered by UEBMI SPA, which meant co-insurance, either through OOP cash or MSAs, constrained medicine over-use. The ‘shrinkage’ of MSAs resulted in the psychological perception of the decreasing consumption ability. Also, there were many competing retail pharmacies, and their distribution meant UEBMI members had retail pharmacy choices, which both constrained price increases and led to price convergence. Finally, the disease spectrum and medicine demand by members remained relatively unchanged.

There were also influencing factors from UEBMI outpatient scheme regulators, which constrained medicine prices and consumption. Although there were differences in the promotion of the 2023 reform in other cities, regulators in Wuhan regarded the claim risk from the UEBMI fund as their main regulatory aim, while also introducing measures to reduce the impact of the new policy on UEBMI’s SPA [26]. Specifically, when purchasing medicine recorded in NRDL, whether prescription or OTC drugs, the pharmacist of retail pharmacies had to strictly comply with policy provisions by checking customer information and prescriptions. While significantly increasing the workload as the number of medicines purchasers increased, the checking of customer information attenuated over-use and over-pricing. The information checking complicated the sales system in contrast to the simple process in the past, increasing the time cost of pharmacist and customer interaction. OTC medicines approved by the National Medical Products Administration were purchased without prescriptions.

The regulations on prescribed medicines purchasing in retail pharmacies did impose some inconvenient restrictions on UEBMI members. For example, the common treatment cycle for gastric helicobacter pylori infection was one month, but the maximum prescription volume did not exceed three days’ usage, which meant members had to make multiple purchases at their retail pharmacy, increasing the indirect cost and decreased the adherence of patients to take their medicine. It also made it impossible for patients to take medicines away when traveling. To control potential UEBMI claim fraud risks, regulators balanced member convenience against medicine hoarding and reselling. Finally, for some serious disease medicines, such as Shaban for the treatment of deep vein thrombosis after hip replacement surgery, human albumin blood products and 2-line mediation for hypertension, paper prescriptions and medical records issued by hospitals were required when purchasing reimbursable medicines in retail pharmacies. These 2023 rules constrained the reform benefits for some members.

Similar to other medical insurance reforms [7, 27], some members did not fully understand the mechanism of the new policy, believing that the ‘shrinkage’ of MSAs was a reduction of welfare when the 30% employer premiums were paid into SPA. This made many members cautious in their medicine consumption. Although the retired members were satisfied with the reform, the currently employed members were less supportive because they were sick less often, could accumulate MSA deposits and large MSA were not available to be passed on to their families. To address the smaller MSAs, family insurance scheme should be considered.

Our study has several limitations. Due to data limitations, we were not able to collect information on the specific medicines in the claims data. Future studies should collect data on specific medicines. Second, ITSA without a control group is applicable for assessing the impact of the reforms, but ITSA cannot provide robust evidence to support the causal relationship between intervention and outcome variables. Third, we collected quantitative data from two retail pharmacies in one Chinese city. Care should be taken in drawing conclusions from our Wuhan study for other cities in China. Given the municipal-province implementation differences across China, pharmacy medicine purchasing studies in other cities should be undertaken.

## Conclusions

From March 2022 to December 2023, we gained unique access to daily UEBMI members’ medicine purchases, covering 34,956 claims at two representative Wuhan pharmacies. The 2023 reform policy of including retail pharmacies in China’s Urban Employee Basic Medical Insurance scheme played a positive role in improving the availability of medicines, benefiting UEBMI members and attenuating the OOP medicine expenditure burden, especially for retired members with higher medical demands. The previous large and unused MSA deposit balance, especially by younger employed workers, was transferred into SPA, which financed the expansion of the UEBMI outpatient scheme to retail pharmacies. The 2023 reforms did not trigger an increase in members’ medicine consumption; promoted access to medicines; attenuated the over-use of hospital pharmacies; and optimized outpatient health resource utilization. Attracting more customers by providing health services transformed pharmacies’ business model away from a singular focus on price competition.

## Data Availability

The datasets used and analyzed during the current study are available from the corresponding author on reasonable request.

## Abbreviations

UEBMI: Urban Employee Basic Medical Insurance
MSAs: Medical Savings Accounts
SPA: Social Pooling Account
OOP: Out-of-Pocket
ITSA: Interrupted time Series Analysis
